# Accelerating the development of bioelectronic medicine: outcomes and innovations from the NIH SPARC initiative

**DOI:** 10.1186/s42234-026-00213-z

**Published:** 2026-09-04

**Authors:** Bhavesh Patel, Victor Pikov, Margot S. Damaser, Jyl Boline, Susan J. Tappan, David P. Nickerson, Matthew P. Ward, Pallavi K. Gunalan, Esra Neufeld, Kip A. Ludwig, Jeffrey S. Grethe, Joost B. Wagenaar, Maryann E. Martone

**Affiliations:** 1 FAIR Data Innovations Hub, California Medical Innovations Institute, San Diego, CA USA; 2 Medipace Inc, Pasadena, CA USA; 3 Department of Biomedical Engineering, Cleveland Clinic Research, Cleveland, OH USA; 4 Advanced Platform Technology Center, Department of Veterans Affairs Medical Center, Louis Stokes Cleveland, Cleveland, OH USA; 5 Informed Minds Inc., Walnut Creek, CA USA; 6 Rock Maple Science, Inc. Hinesburg, Hinesburg, VT USA; 7 Auckland Bioengineering Institute, University of Auckland, Auckland, New Zealand; 8 Purdue University, West Lafayette, IN USA; 9 University of Southern California, Los Angeles, CA USA; 10 Foundation for Research on Information Technologies in Society (IT’IS), Zurich, Switzerland; 11 University of Wisconsin-Madison, Madison, Wisconsin USA; 12 Department of Neurosciences, School of Medicine, University of California San Diego, La Jolla, CA USA; 13 Department of Biostatistics, Epidemiology and Informatics, University of Pennsylvania, Philadelphia, PA USA

**Keywords:** Bioelectronic Medicine, Autonomic Neuromodulation, Device, Data, Consortium, Vagus

## Abstract

The NIH Common Fund’s SPARC (Stimulating Peripheral Activity to Relieve Conditions) program was launched in 2015 to catalyze bioelectronic medicine by advancing foundational knowledge of the autonomic nervous system (ANS) and enabling clinical translation of novel bioelectronic medicines. While the program officially ended in 2025, activities related to the program are still ongoing through no-cost extensions, data sharing, and derivative efforts. We provide here an overview of the major outcomes of SPARC under its four focus areas: Anatomical and Functional Mapping, Technology Development, Translational Research, and Data/Modeling Infrastructure. In the Anatomical and Functional Mapping focus areas, teams generated cross-species, cross-organ ANS connectivity datasets, detailed nerve anatomy, along with detailed maps and connectivity resources to support query, visualization, and hypothesis-generation. Under the Technology Development focus area, teams developed new tools for recording and stimulating autonomic pathways and built computational pipelines for realistic neuromodulation simulations. Within the Translational Research, teams conducted pre-clinical and clinical proof-of-concept studies and used prize-driven efforts to accelerate clinically ready neuromodulation solutions. Data/Modeling Infrastructure teams built a durable open-science ecosystem enabling FAIR sharing, exploration, and reuse of SPARC datasets, models, and workflows by the broader community. Together, SPARC’s integrated approach has lowered barriers to precision neuromodulation research and established reusable resources to sustain the field beyond the program.

## Introduction

The NIH Common Fund’s SPARC (Stimulating Peripheral Activity to Relieve Conditions) program, established in 2015, sought to accelerate the development of therapeutic devices that modulate electrical activity in the autonomic nervous system (ANS) to improve internal organ functions and suppress dysfunction. This therapeutic strategy is known as bioelectronic medicine or autonomic neuromodulation (Peeples 2019). The ANS, consisting of parasympathetic and sympathetic nervous systems, controls every internal organ in the body besides the brain. Therefore, the clinical impact of bioelectronic medicine can be extensive, covering many types of health issues such as neurological, cardiovascular, urological, gastrointestinal, respiratory, hepatic, renal, gynecological, autoimmune, infectious, metabolic (obesity and diabetes), and potentially even oncological.

Chronic diseases are a leading cause of morbidity, with 43% of people in North America experiencing two or more chronic diseases, which negatively impact the quality of life of affected individuals (Samiei Siboni et al. 2019; Chowdhury et al. 2023). Therefore, adding bioelectronic medicine to the regimen of available therapies could considerably reduce morbidity and improve the quality of life of patients with chronic diseases, especially for those intolerant or resistant to medications, or seeking alternatives to irreversible surgical procedures. In addition to long-lasting efficacy, bioelectronic medicine offers additional advantages over medications by providing adaptable control of therapy dosing based on symptom mringonito (Mylavarapu 2023), an "implant it and forget it" approach achieving nearly 100% therapy compliance (Woo et al. 2023), and a lower risk of side effects due to its targeting of organ-specific nerve branches (ikov 2023). The vagus nerve has been an obvious target given that it is the longest nerve in the body and plays a key role in the reflex regulation of most internal organs.

While showing great promise for broad applicability, relatively few bioelectronic medicine approaches were routinely used in clinical care before 2015 due to several gaps in knowledge and technology (Birmingham 2014). These include a lack of details of how peripheral nerve pathways innervate internal organs, a limited understanding of the neural signaling patterns controlling organ function, and the absence of neural interfaces optimized for small-diameter autonomic nerves (<500 µm). Therapeutic feasibility was also uncertain due to open questions around how anatomical variability across subjects impacts neuromodulation efficacy, how to selectively target complex nerves, and how neural encoding and organ-functional control are realized. Moreover, the neuroanatomical relevance of animal models was unclear, particularly pertaining to on- and off-target effects of clinical neuromodulation. Robust tools to assess local and downstream target engagement in animal models were not available. Finally, effective and standardized data sharing practices were lacking, which prevented novel discoveries from secondary data reuse.

The SPARC program, initiated in 2015 by the National Institutes of Health (NIH) in the United States of America (USA), brought together a diverse group of neuroscientists, engineers, clinicians, data scientists, and other experts to address these gaps. The overarching goal of the program was “to catalyze development of new or more efficacious therapies utilizing neuromodulation of end-organ system function.” The SPARC program was structured around four interdependent focus areas: Mapping, Technology, Translational Research, and Data and Resources. In Phase 1 (2015–2021), these initiatives worked together to advance understanding of the ANS, develop neuromodulation tools, and make all outcomes openly available and FAIR (Findable, Accessible, Interoperable, Reusable) (Wilkinson 2016) via the SPARC Portal (https://sparc.science) with support from the SPARC Data and Resource Center (DRC). Launched in 2022, Phase 2 of the program continued these four focus areas with a targeted emphasis on the human vagus nerve, aiming to generate detailed anatomical and functional maps, build open-source devices, and enable new therapeutic strategies. While the program officially ended in 2025, activities related to the program are still ongoing through no-cost extensions, data sharing, and derivative efforts.

Overall, the program awarded over $350M to close to 100 projects that generated 500+ publications and 300+ datasets. As the program is coming to an end after over 10 years of activity, we highlight some of its achievements and the direction it has set for the bioelectronic medicine field. The companion paper led by our NIH colleagues provides more background and details about the SPARC Program and its impact. Here, we provide an overview of the major scientific outcomes and innovations across the four focus areas (also summarized in Table 1).

**Table 1:** Notble outcomes in Phase 1 across the SPARC priority areas organized by focus area (Mapping, Technology, Translation, Data)

**Team location**	**Outcome**
*Mapping*	
University of Central Florida and Thomas Jefferson University	Comprehensive 3D atlas of the rat intrinsic cardiac nervous system (ICN) at the single-cell resolution to characterize the distribution patterns of intrinsic cardiac neurons between individuals and across sexes (Leung 2021, 2023, 2020)
University of Wisconsin-Madison and Duke University	Combined microdissection, histology, and immunohistochemistry to provide data on key features across the cervical vagus nerve in a swine model and then compared it to other animal models (mouse, rat, dog, non-human primate) and humans (Settell 2020; Ludwig 2022, 2022).
University of Melbourne	Used immunohistochemical immediate-early gene (IEG) markers to map neuronal activity in lumbosacral spinal cord after stimulating intense micturition in awake, unrestrained female and male rats, and showed sex difference (Wiedmann et al. 2021; Keast 2020; Bertrand et al. 2020).
Purdue University	Conducted a foundational mapping project that advanced the field toward precision gastric neuromodulation through intensive pre-clinical and clinical investigations including mapping vagal-gastric circuit at high resolution, and defining innervation maps to guide simulation site and parameter selection for precision gastric neuromodulation. (Powley 2019, 2021; Lu 2022; Tan 2021; Ward 2020).
*Technology*	
Case Western Reserve University	Developed bladder and bowel monitors to wirelessly transmit organ function in conscious ambulating animals for potential closed loop or triggered stimulation (Damaser 2023, 2023, 2022, 2022; McAdams 2018, 2018).
California Medical Innovations Institute	Developed Fecobionics, a simulated feces that provides an objective, safe, and low-cost assessment of defecatory function by measuring a variety of parameters during defecation of the device including pressures, bending, and shape changes which can be used for improved diagnosis of anorectal disorders including constipation and fecal incontinence (Wang et al. 2021; Gregersen 2022; Wang 2023, 2023).
Duke University	Developed ASCENT (Automated Simulations to Characterize Electrical Nerve Thresholds), a pipeline for sample-specific computational modeling of electrical stimulation of peripheral nerves which is used to virtually design experimental and clinical neuromodulation interventions (Musselman et al. 2021; Marshall 2023, 2023, 2023; Musselman 2023, 2023, 2023, 2023; Davis 2023).
*Translation*	
Indiana University	Used a canine model of atrial fibrillation (a type of arrhythmia) to compare the effectiveness of VNS and sympathetic nerve stimulation in slowing down the ventricular rate and determined that high-intensity sympathetic nerve stimulation was more beneficial for treating atrial fibrillation (Kusayama 2021; Jiang 2018).
University of Michigan	Used a feline model of overactive bladder to compare the effectiveness of open-loop sacral nerve stimulation (SNS) and closed-loop SNS, where bladder sensory feedback was recorded from dorsal root ganglia, and demonstrated superior performance of closed-loop SNS (Ouyang 2022).
Johns Hopkins University	Applied spinal cord stimulation at the thoracic level in a canine model of gastroparesis and observed normalization of gastric motility along with suppression of sympathetic overactivity (Zhang et al. 2019).
*Data*	
The SPARC Data Resource Center (DRC) - IT’IS Foundation, University of Auckland, University of California San Diego, University of Pennsylvania	Developed the SPARC Portal which was launched at the International Society of Autonomic Neuroscience (ISAN) meeting in July 2019 and has been actively expanded since (SPARC Portal n.d.). In a move towards sustainability for the SPARC Portal, DRC PIs expanded focus from the ANS to the PNS and developed resources and materials for investigators to submit their data to SPARC in preparation for the NIH data sharing mandate in January 2023. The DRC now supports non-SPARC investigators and continues to develop new resources and methods to support these new members of the SPARC community.
University of Pennsylvania	The datasets on the SPARC Portal are hosted through the Pennsieve platform, which was initially developed at Blackfynn Inc. In 2021, the platform was made open-source and further development and maintenance of the platform and associated projects were transferred to the University of Pennsylvania.
University of California San Diego	The University of California San Diego provides the semantic foundation that makes SPARC data FAIR by developing standards, curation workflows, and knowledge infrastructure that integrate experimental data across the autonomic nervous system. UCSD established the SPARC Dataset Structure (SDS), a standardized organization and metadata framework that captures rich experimental metadata and ensures consistent submission, validation, and publication of SPARC datasets (Bandrowski 2021). UCSD also develops and maintains the SPARC Connectivity Knowledge base of the Autonomic Nervous system (SCKAN), a knowledge graph that integrates anatomical and neural connectivity knowledge across species to support the exploration of the autonomic nervous system (ANS) and end organ connectivity (Imam 2025).
University of Auckland	The University of Auckland develops the spatial framework that enables SPARC datasets, anatomical knowledge, and computational models to be integrated within common anatomical coordinate systems (Osanlouy 2021). Central to this effort are anatomically accurate 3D organ scaffolds, generated using finite element-based material coordinate systems, which provide a common spatial framework for organs such as the heart, lungs, bladder, stomach, and colon while accommodating anatomical variation across individuals and species. Complementing these scaffolds are interactive mapping user interfaces, 2D flatmaps, and 3D anatomical viewers that enable researchers to navigate neural pathways, anatomical structures, and associated datasets through intuitive spatial exploration rather than traditional file-based search.
IT’IS Foundation	The computational models on the SPARC Portal are hosted through o^2^S^2^PARC, an open, online-accessible, cloud-based platform that supports collaborative, reproducible, sustainable, and FAIR elaboration, sharing, publication, execution, and deployment of simulations, data analysis, expert workflows, and applications (Osparc-Simcore: Osparc-Simcore Simulation Framework n.d.). For example, the interoperability, reusability, and collaborative modeling capabilities of o^2^S^2^PARC were leveraged to combine AI-based creation of personalized spinal cord models from medical image data, electromagnetic simulations, neural dynamics modeling, and efficient optimization techniques to design and guide research on a bioelectronic implant for bladder control.

### Anatomical and functional mapping

The SPARC mapping initiative supported the creation of new anatomical and functional connectivity datasets aimed at driving hypothesis generation. Key areas of investigation included the coursing and branching of peripheral nerves, the distribution of axon terminals, nerve-organ synapses, the cross-sectional organization of nerves, the functional effects of firing patterns on organs, and the relevance of particular animal models to human systems.

#### Comprehensive cross-species and cross-organ mapping of the ANS

In Phase 1 (2015-2021), the mapping initiative focused on five major organ systems: the heart, lungs, stomach, colon, and bladder. Funded studies initially characterized the neural circuitry under normal/healthy conditions using cutting-edge tools such as optogenetics and other genetic approaches to label and target specific neural tracks. In the later years of Phase 1, studies focused on large animal and disease models to better inform translational neuromodulation studies. Additional projects mapped the innervation of less-studied organs, including the spleen, pancreas, esophagus, kidney, liver, adipose tissue, bone, and bone marrow. In a joint effort with the NIH HEAL (The Helping to End Addiction Long-term) Initiative, some projects studied specific peripheral circuits in visceral pain signaling.

Amongst notable outcomes of the SPARC mapping initiative in Phase 1 was the development of a comprehensive 3D atlas of the rat intrinsic cardiac nervous system (ICNS) at single-cell resolution. Conducted by researchers at the University of Central Florida and Thomas Jefferson University, this work characterized the distribution patterns of intrinsic cardiac neurons between individuals and across sexes, offering a rich dataset for comparative and translational research (Leung 2021, 2023, 2020). The researchers developed a computational approach to bridge the gap between widely available molecular-level gene expression and sparse cellular-level electrophysiology to determine the functional role of the ICNS in cardiac regulation and pathology (Gupta 2025). They used single-cell omics data to build computational models to simulate biological systems at various scales, integrating single-cell data with physiological information to generate organ-specific models, which can then be assembled to generate multi-organ systems pathophysiological models and even patient-specific predictive models (Manchel et al. 2024). A strategy was also developed to use computational models to organize and guide experiments aimed at harnessing the cardiovascular health benefits of differential activation of the fast and slow lanes of the vagus nerve (Gee 2024).

Investigators from the University of Wisconsin-Madison and Duke University combined microdissection, histology, and immunohistochemistry to analyze and compare the structure of the cervical and subdiaphragmatic vagus nerve in human, swine, and rat (Settell 2020; Ludwig 2022, 2022). Significant species differences were found, including a greater morphological variability across human samples compared to swine and rats (Pelot 2020). The investigators then went on to use this data to create a model to simulate vagus nerve stimulation (VNS) in humans, pigs, and rats. The human models successfully captured inter-individual variability for both acute and chronic implants, while swine models consistently captured the range of*in vivo*thresholds across all measured nerve and physiological responses (Musselman et al. 2023). The models in human, pig, and rat were used to quantify nerve responses across species and simulate translation of VNS therapies via either recycling or linear scaling of stimulation parameters. The investigators found that applying the same VNS parameters across individuals within a species or across species produced a large range of nerve responses, highlighting the need for systematic approaches to select stimulation parameters that account for individual- and species-specific differences in nerve responses to stimulation (Musselman et al. 2025).

Investigators from the University of Melbourne identified sex differences when they looked at immunohistochemical immediate-early gene (IEG) markers to map neuronal activity in the lumbosacral spinal cord after stimulating intense micturition in awake, unrestrained female and male rats (Wiedmann et al. 2021; Keast 2020; Bertrand et al. 2020). They quantified afferent innervation in the lumbosacral spinal cord using intramural microinjection of cholera toxin and established a non-surgical method to visualize and map myelinated afferents in the bladder in rats using an adeno-associated virus (Fuller-Jackson et al. 2021). This virus primarily transduced myelinated dorsal root ganglion neurons, enabling identification of regional distribution of myelinated axon endings within the muscle and lamina propria of the bladder, as well as myelinated afferents within the pelvic nerve and lumbosacral spinal cord (Wiedmann et al. 2024). They also developed a deep learning based high-throughput processing pipeline for automated segmentation of unmyelinated fibers and validated it using scanning electron microscopy of nerves in rat bladders (Plebani 2022). With other SPARC investigators, they employed spatial statistics and point process models to describe the spatial arrangement of axons to enable quantitative comparisons of similarities between these arrangements in vagus and pelvic nerve cross-sections (Shemonti 2023). This novel technique enables comparisons between sexes, different ages, different species, and in a variety of pathologies.

Through a foundational mapping project that led to over 40 publications and patents (RePORT ⟩ RePORTER n.d.), Dr. Terry Powley and team advanced the field toward precision gastric neuromodulation through intensive pre-clinical and clinical investigations. This 5-year project centered on 1) mapping the relevant vagal–gastric circuitry at high resolution (Powley 2019, 2021; Lu 2022), 2) defining innervation maps that defined stimulation site/parameter choices (Lu 2022; Tan 2021; Ward 2020), and 3) disseminating datasets and measurement approaches to enable reproducible, closed-loop-ready device development for stomach disorders.

**Fig. 1:**
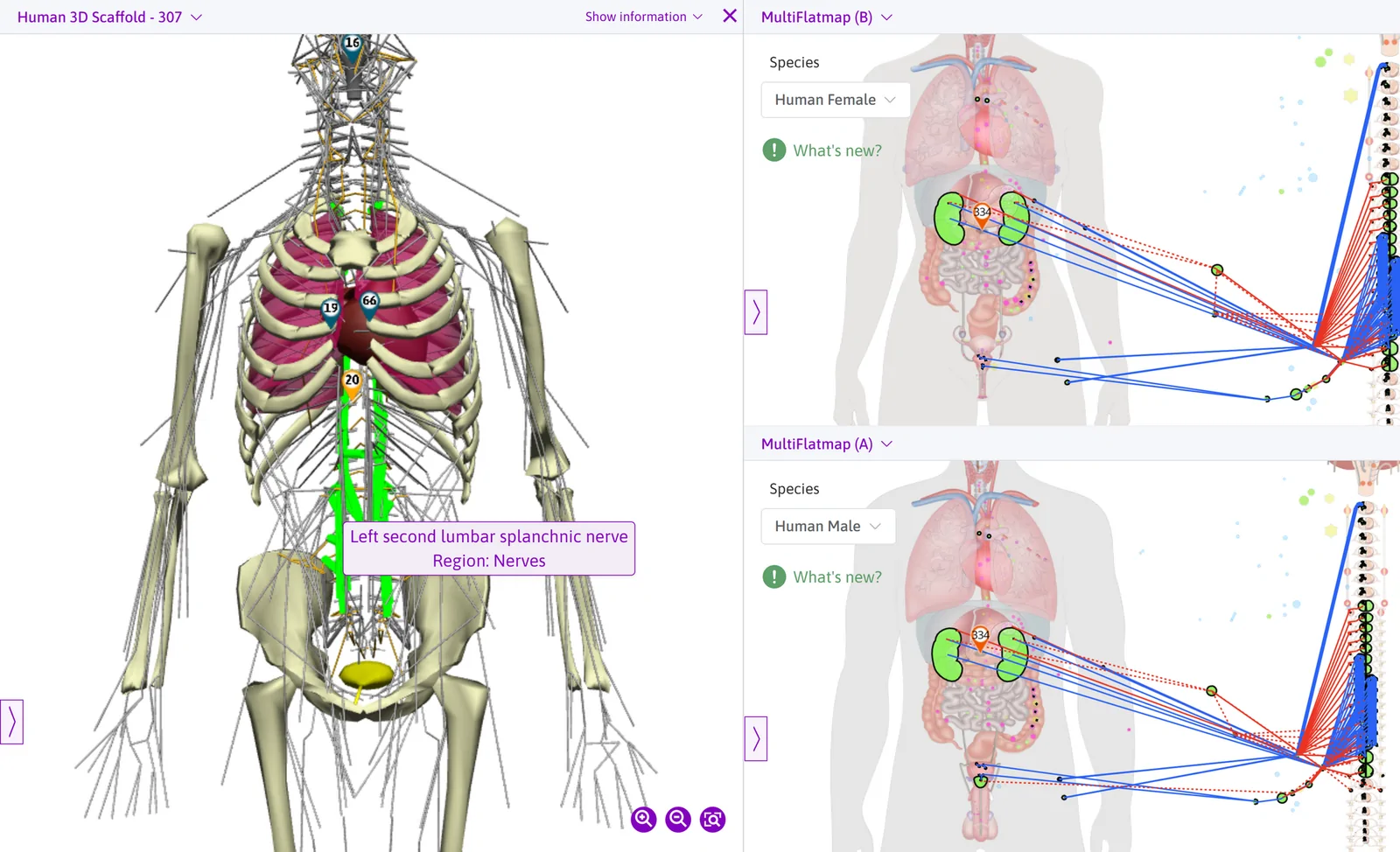
Illustration demonstrating how selecting a nerve in the whole-body scaffold (the “left second lumbar splanchnic nerve” in this example) queries SCKAN for the neuron populations travelling via that nerve and selects them on the male and female Human anatomical connectivity flatmaps. Visit https://sparc.science/apps/maps?id=30fcea02 to reproduce this view of the maps. Note that some features in the whole-body scaffold map have been hidden to aid visibility of the selected nerves

Overall, efforts from the mapping initiative in Phase 1 supported the development of the first anatomical connectivity maps of the ANS across various species, including humans (both male and female), rats, mice, pigs, and cats. A unique resource was created by members of the SPARC DRC to capture SPARC investigators’ expert knowledge about ANS connectivity in a machine-computable knowledge base that allows query and reasoning. This resource is the SPARC Connectivity Knowledge Base (SCKAN) (Imam 2025), which describes connections at the neuron population level, specifying the locations of cell bodies, axons, axon terminals, and synapses, and synaptic targets via the Neuron Phenotype Ontology (Gillespie et al. 2022). SCKAN underlies the anatomical connectivity maps on the SPARC Portal as well as a new functional connectivity flatmap that provides a visualization of semantic connectivity. Additionally, a comprehensive 3D human whole-body scaffold has been established with integrated musculoskeletal, vasculature, nervous systems, and organs. Interactive implementations of these maps are accessible on the SPARC Portal at https://sparc.science/apps/maps (Fig. 1).

#### Focus on the human vagus nerve

In Phase 2, the SPARC mapping initiative has focused on the human vagus nerve, the autonomic nerve responsible for parasympathetic control over the majority of internal organs. These ambitious research studies are producing massive amounts of diverse data, giving new insights into the detailed structure (Reconstructing Vagal Anatomy, REVA) and function (VNS Endpoints from Standardized Parameters, VESPA) of the vagus nerve.

In REVA, investigators at Feinstein Institute for Medical Research, Case Western Reserve University, and Duke University utilized gross dissection and multi-scale imaging of the vagus nerve from human cadavers to generate high-resolution connectivity profiles with special focus at predefined points of interest (branching points and potential sites of neuromodulation device interface) (Joseph 2025; Buyukcelik 2023). Morphologic metrics, including nerve diameter, fascicle count, fascicle area, fascicle diameter, and perineurium thickness were compared using a neural network approach for interindividual differences. This work characterizes how the axons and fascicles within the vagus nerve are organized along its length and branches, and quantitatively describes the extent of anatomical variability among individuals. The researchers also developed methods to automate microscopic tractography on 3D-MUSE datasets of peripheral nerves, demonstrating in a vagus nerve sample that tractography readily identified that subsets of fibers from 4 distinct fascicles merge into a single fascicle ~5mm along the nerve's length (Kolluru 2024).

VESPA investigates the functional connectivity of the human vagus nerve and the physiological effects of altering vagus nerve activity. This clinical study is capturing multi-organ readouts of standardized stimulation parameters in patients implanted with vagus nerve stimulation (VNS) devices to discover how best to stimulate nerve fibers for specific therapeutic effects. Creating more precise and detailed anatomical and functional maps of the vagus nerve will help scientists identify how to stimulate nerve fibers safely and effectively for bioelectronic medicine therapies. Some of these groundbreaking datasets are already offered to the public on the SPARC Portal, and new technologies built by the DRC to highlight and support these datasets are also coming online.

### Technological Innovations

The SPARC technology initiative fostered the development of cutting-edge technologies for studying and interfacing with the ANS and internal organs. The technological innovations from this initiative supported the other SPARC initiatives by enabling new ways to study and stimulate the ANS.

#### Cutting-edge technologies

In Phase 1, SPARC supported the development of cutting-edge technologies across a wide array of disciplines, including photonics, system engineering, virology and genomics, device design and manufacturing, surface chemistry, tissue engineering, and more. A major focus of this phase was on developing tools to simulate innervation circuits and neural activation thresholds, as well as sensors capable of detecting changes in nerve activity and organ function. The vision was for these tools to be integrated into closed-loop systems that inform when and how stimulation should be applied to restore physiological balance.

**Fig. 2:**
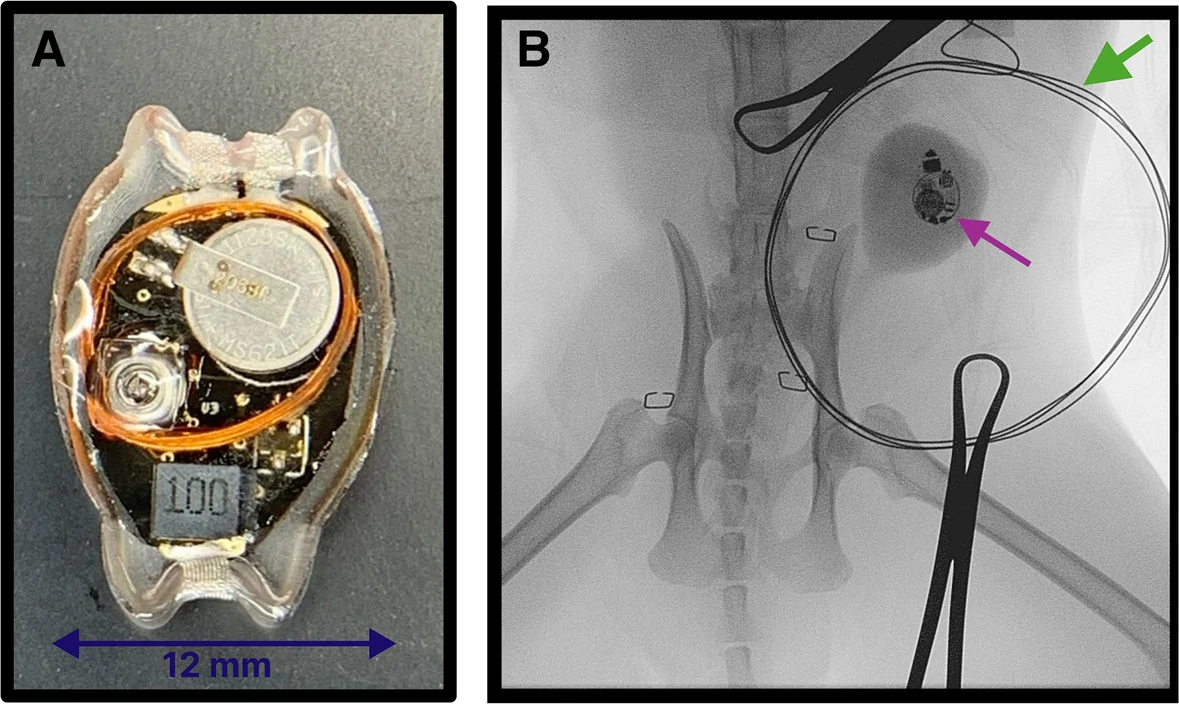
The Urological Monitor of Conscious Activity (UroMOCA) sensor (**A**) which is implanted suprapubically into the lumen of a cat bladder where it resides for up to 30 days (**B**). The double headed arrow in A indicates that the width of the UroMOCA is 12 mm. The height is 18 mm. The purple arrow in B indicates the UroMOCA in the bladder visualized on a CT scan. Green arrow in B indicates the external antenna used to receive wireless data transmission from the implanted UroMOCA sensor

Several of the technologies developed through this initiative in Phase 1 have since entered clinical trials, received patents, obtained FDA approval, or become widely used in neuromodulation research. For example, a team at Cleveland Clinic, Case Western Reserve University, and the Louis Stokes Cleveland VA Medical Center developed the Urological Monitor of Conscious Activity (UroMOCA) sensor system to measure bladder pressure and volume for up to 30 days in conscious ambulating animals (Fig. 2) (Damaser 2023, 2023, 2022, 2022; McAdams 2018, 2018; Majerus 2024). The investigators used this novel system to assess the impact of different frequencies of sacral nerve stimulation (SNS) on lower urinary tract function in ambulating animals, demonstrating that high-frequency stimulation can reduce pelvic floor and urethral contraction, enabling bladder emptying (Han 2025). Since experiments with conscious ambulating animals extending over many days and weeks generate a great deal of data, the investigators developed algorithms aimed at automated detection of important features and events (Zareen 2024). The group developed a spin-off technology to assess colon activity in conscious ambulating animals, the Colonic Monitor of Conscious Activity (ColoMOCA) (Majerus 2023). The bladder technology has been licensed to Bright Uro, Inc who have obtained FDA approval for the sale of their initial device, the*Glean*, for improved diagnosis of lower urinary tract dysfunction in people.

Researchers at the California Medical Innovations Institute developed Fecobionics, a simulated feces that provides an objective, safe, and low-cost assessment of defecatory function by measuring a variety of parameters during defecation of the device, including pressures, orientation, bending, and shape changes. The wireless device is insertable and distinctly different from catheter-based manometry systems and radiology systems, e.g., Fecobionics measures axial pressures along the defecatory trajectory during expulsion whereas manometry systems measure radial pressure at fixed positions. The simultaneous measurement of multiple parameters is the foundation for computation of complex parameters such as friction force and defecation indices. The Fecobionics device is FDA-cleared and can be used for improved diagnosis of anorectal disorders, including constipation and fecal incontinence. Furthermore, the device can be used for monitoring of therapies and for biofeedback therapy of patients with defecatory disorders, i.e., for retraining of pelvic floor coordination and for desensitizing rectal hypersensitivity (Gregersen 2022, 2022, 2023, 2025).

Duke University researchers developed ASCENT (Automated Simulations to Characterize Electrical Nerve Thresholds), a powerful open-source computational pipeline for anatomically realistic modeling of electrical stimulation of peripheral nerves (Musselman et al. 2021). Since its original publication, updates to ASCENT include: 1) integration of the PyFibers pipeline for Python-based simulation of multi-compartment models of nerve fibers, replacing the prior HOC code(Marshall 2025); 2) added capability to model neural recordings(Peña et al. 2024), in addition to neural stimulation and block; and 3) redesigned documentation for improved usability hosted on Read the Docs (Welcome to ASCENT’s documentation! — ASCENT v1.5.0 documentation n.d.). To build the 3D model nerve, users can supply image-based, sample-specific inputs (e.g., segmented histology) or can use ASCENT to define a synthetic nerve morphology. Similarly, users can use built-in or customized electrode geometries and stimulation parameters. ASCENT thus enables design of both experimental and clinical neuromodulation interventions, predicting how different electrode geometries, electrode placements, and stimulation parameters interact with multi-scale nerve anatomy and biophysics to modulate neural activity. Researchers can explore optimal stimulation strategies in silico before proceeding to in vivo and clinical studies, accelerating the development of targeted therapies while reducing experimental costs and risks.

Multiple studies used ASCENT to analyze and design neuromodulation therapies, including validated models of human, pig, and rat vagus nerve stimulation (Blanz 2023; Musselman 2023), inter-species translation of stimulation parameters (Musselman et al. 2025), design of stimulation parameters for selective activation (Hussain et al. 2024; Rossetti 2025), integration of multi-modal imaging data for model parameterization (Pelot 2025), anatomical and electrical properties that result in lower thresholds for fibers in smaller fascicles (Davis 2023), and cuff vs. intraneural electrodes for recording unmyelinated fiber activity (Davis 2023). In addition to its open-source code and documentation, ASCENT simulations can be executed on the o2S2PARC platform (Marshall 2023, 2023). ASCENT also includes documentation (Welcome to ASCENT’s documentation! — ASCENT v1.5.0 documentation n.d.) for reporting ASCENT-based methods and a utility for generating standardized ASCENT-based datasets, as reflected by numerous datasets on the sparc.science data repository platform (Marshall 2025; Davis 2023; Musselman 2025, 2023, 2023; Pelot 2024). Importantly, development of ASCENT and other neural stimulation models informed actionable guidance—from project initiation through publication—for scientific code, models, and software that are replicable and reusable.

**Fig. 3:**
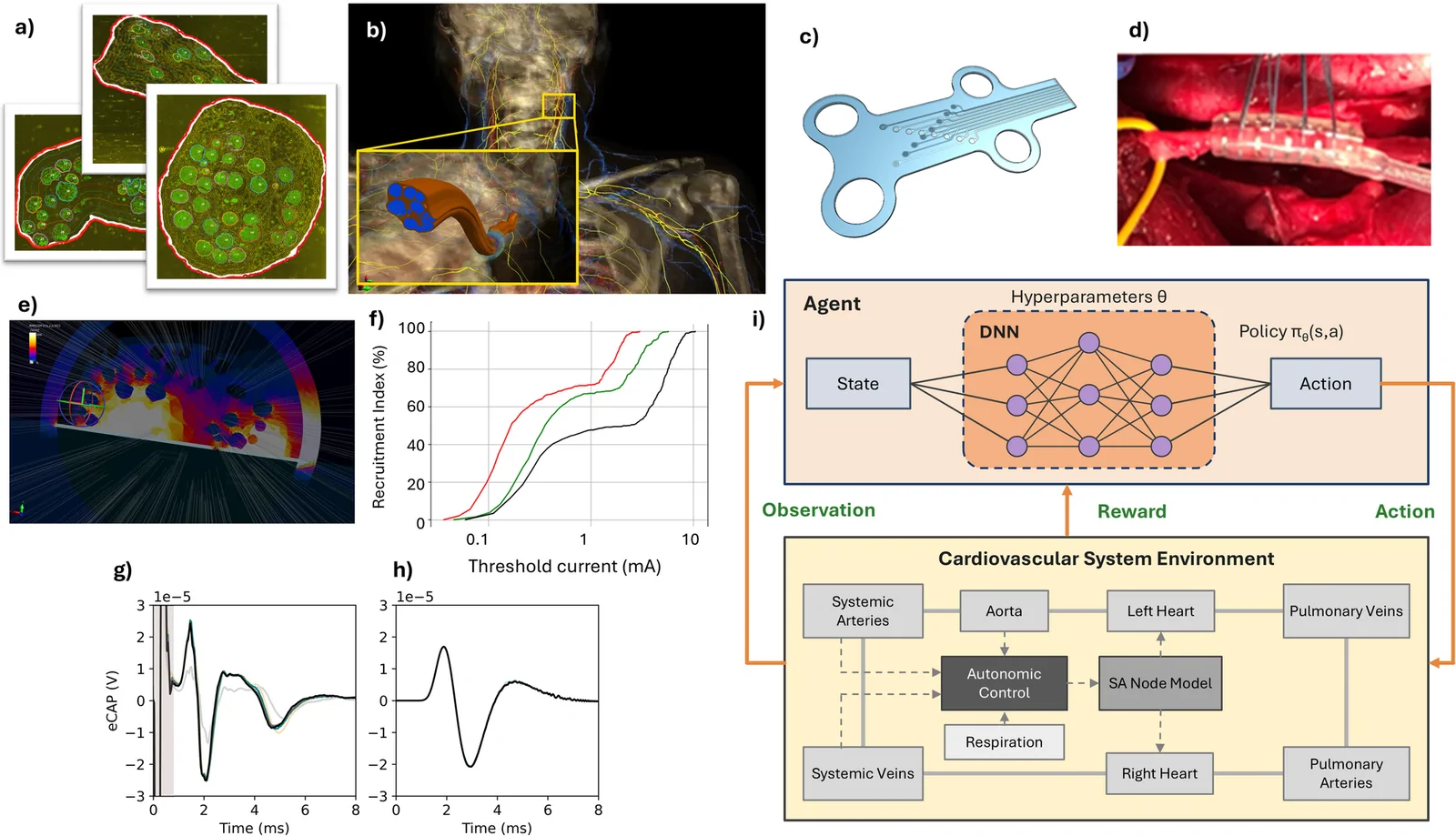
Model predictive control for a closed-loop vagus nerve stimulation implant to restore cardiovascular control to transplant patients; (**a**) SPARC histological data is segmented and (**b**) extruded along pre-traced nerve trajectories in a NEUROCOUPLE model, (**c**) before integrating a stimulating TIME and a recording cuff electrode in the nerve model, in accordance with (**d**) the experimental setup. Heterogeneous populations of electrophysiological fiber models are integrated and (**e**) exposed to a simulated electric field (**f**) to predict recruitment curves. The model is validated by comparing (**g**) measured compound action potential signals (**h**) to simulated ones. **i** The neural interface model is coupled to a model of cardiovascular regulation, and a deep neural network (DNN) is trained to predict cardiovascular responses to stimulation. That DNN serves as internal model for model predictive control, enabling regulation of heart rhythm and blood pressure; the entire pipeline was implemented on SPARC’s platform for open and collaborative computational modeling – o^2^S^2^PARC

The IT’IS Foundation leveraged similar hybridized electromagnetic–neuron electrophysiology modeling using the computational life sciences platform Sim4Life (Sim4Life. n.d.), on o^2^S^2^PARC (SPARC’s modeling and model publication platform (Guidon 2025); seeData and Modeling Infrastructure section) and combined it with dosimetric assessment, induced heating simulation, and electrochemical damage predictors to perform *in silico *safety assessment. As part of the effort required to achieve regulatory-grade modeling suitable as regulatory evidence, they executed a comprehensive prediction uncertainty quantification study, employing the surrogate-modeling-based uncertainty quantification module in o^2^S^2^PARC (Neufeld 2025; Neufeld et al. 2025). Multiple major manufacturers of bioelectronics implants are now leveraging this pipeline for submissions to the U.S. Food and Drug Administration (FDA). Together with the Medical University in Vienna, the pipeline was also used to model a restorative neural interface on the cardiac vagus nerve branch for the purpose of restoring cardiovascular control in heart transplant patients (Fig. 3). It was combined with a detailed model of cardiovascular regulation and used to train a deep neural network (DNN) for model predictive closed-loop control (Haberbusch 2023). Its performance was validated in rabbit heart experiments, demonstrating superior control accuracy and speed.

#### Toward open-source and clinical-grade neuromodulation devices

In Phase 2, the technology initiative aims to develop interoperable open-source modules (e.g., hardware, firmware, and software elements) that can be combined into therapy-specific implantable devices for new exploratory clinical neuromodulation studies in the ANS. The open-source libraries include regulatory documentation, device technical specifications, hardware designs, manufacturing and assembly processes, testing procedures, validation reports, and firmware and software code that are designed in compliance with the FDA regulatory requirements to reduce the financial, personnel, and regulatory burden in the development of new clinical-grade devices for bioelectronic medicine. This open-source ecosystem will reduce the barrier to entry into bioelectronic medicine research and will speed the development of new therapies.

To this end, investigators at Case Western Reserve University established the Cleveland Open Source Modular Implant Innovators Community (COSMIIC) and investigators at the University of Southern California, Medipace Inc, and Med-Ally established the Center for Autonomic Recording and Stimulation Systems (CARSS) to develop an open-source, modular network of active implantable devices for use in early feasibility human research and to provide ongoing support for this technology through a vibrant, sustainable community of active users. CARSS and COSMIIC teams are funded by the SPARC Human Open Research Neural Engineering Technologies (HORNET) Program to create an open-source ecosystem for collaborative research in bioelectronic medicine (Baldwin 2025; Baldwin et al. 2025). The SPARC Portal has recently been expanded to include a method to publish these devices. The key technologies developed by the COSMIIC and CARSS teams are described below.

##### Neural interface leads for nerve and muscle stimulation

**Fig. 4:**
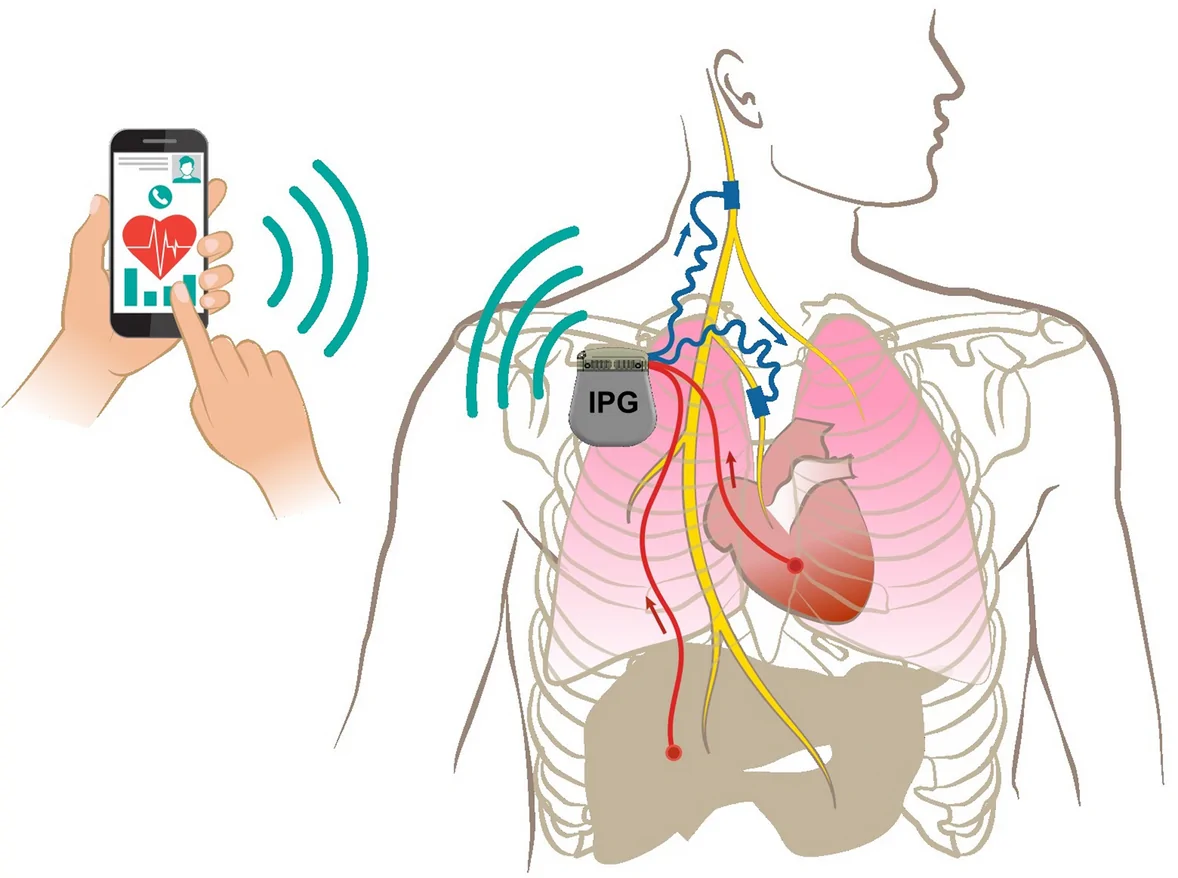
Schematic illustration of the CARSS OpenNerve system, which includes the IPG and four attached leads: two with the cuff electrodes for nerve stimulation (shown in blue) and two for sensing from various internal organs (shown in red). The IPG communicates with an external controller, such as mobile phone, using Bluetooth Low Energy

The HORNET program has made significant progress in utilizing novel shapes, materials, and surgical tools that minimize the risk of nerve trauma, connective tissue scarring, and/or skin erosion. The OpenNerve device, developed by CARSS, includes two cuff electrode types (large and small) for VNS (see blue-colored leads in Fig. 4) and a linear electrode array for SNS (not shown). The large VNS cuff is a full cylinder with a diameter from 1 to 3.5 mm, made from silicone rubber, for interfacing with the cervical or abdominal vagal nerve trunk or with the dorsal roots in the spinal cord (Larauche 2026). It has two circumferential platinum-iridium foil electrodes (1 mm wide and spaced 6 mm apart). The small VNS cuff is shaped either as a full cylinder or a 1.5-turn helix with a diameter below 1 mm, made from a thin-film polymer (Parylene C), for interfacing with small vagal nerve branches. The thin-film polymer is thermoformed, allowing the cuff to self-close and self-size (Elyahoodayan 2024; Rezard 2025). It has two electrodes coated with platinum, platinum-iridium, or other alloys. Since small vagal nerve branches are typically located deep inside the body, the small cuff comes with a novel surgical tool for its laparoscopic placement. The SNS linear electrode array contains four platinum-iridium ring electrodes (3 mm wide and spaced 3 mm apart) mounted along with four tissue anchors (tines) on the polyurethane lead, the same as in the commercial InterStim leads from Medtronic (Persu et al. 2021). The Networked Neuroprosthesis (NNP) device, developed by COSMIIC, includes two leads for muscle stimulation: intramuscular and epimysial (Makowski 2021). The intramuscular linear lead includes a monopolar 2-mm-wide ring electrode and a barbed anchor at the end. The epimysial lead includes two 3.3-mm-diameter platinum-iridium disk electrodes encased on a flat surface, formed by silicone rubber reinforced with polyester mesh. In addition to innovative electrode designs, the HORNET teams also developed novel electronic circuits for enabling blocking of neural activity. The OpenNerve device added the circuit for applying regular VNS pulses to one cuff, while simultaneously applying low-frequency sine waves (1–10Hz) to another cuff placed on the same vagus nerve to provide a unidirectional nerve block. In parallel, COSMIIC’s device added the circuit for applying high-frequency pulses (10 kHz and higher) to provide bidirectional nerve block (Bender 2026).

##### Leads for closed-loop biosensing and control

Addition of biosensors to the implantable bioelectronic medicine device is important for enabling the closed-loop therapy that can be started, stopped, or modulated based on changes in the disease state (e.g., hypoxia, increased inflammatory disease activity) and/or in the physiological state (e.g., postprandial gastrointestinal activity, bladder stretching, adrenal gland activation, sleep). These sensors can also be used to detect excessive stimulation levels, for example, to avoid off-target VNS effects on cardiovascular and respiratory systems. Sensing of these diseases and physiological states requires various types of sensors, including electrical, mechanical, and electrochemical. The OpenNerve device includes all of these sensor types as the optional leads (see red-colored leads in Fig. 4) (Baldwin 2025). Electrical sensors include the electrocardiographic (heart rate, heart rate variability, respiratory rate), electromyographic (somatic and smooth muscle activity), and electroneurographic (nerve activity, including evoked compound action potentials) as well as electrical impedance sensing (detection of failures in VNS and SNS leads or monitoring bioimpedance in various internal organs) based on the same VNS/SNS electrodes described in the earlier section (Larauche 2026). Mechanical sensors include the accelerometer for body position and fast organ movement (physical activity, heart and arterial pulsation),and capacitive strain sensor for slow organ movement (bladder stretching, diaphragm movement, intestinal peristalsis) (Huang 2025). Enzyme-free electrochemical sensors include the sensors for acetylcholine (parasympathetic activation), catecholamine (sympathetic activation), pH (infection, tumor detection, gastroesophageal reflux), and temperature (inflammation, organ activation) (Larauche 2026). More implantable sensors may be added to the OpenNerve device in the future, including the sensors for oxygenation (hypoxia in sleep apnea), pressure (pulmonary artery pressure monitoring in heart failure), and continuous glucose monitoring (type 1 and 2 diabetes).

##### Implantable systems

OpenNerve device features a modular design and includes the implantable pulse generator (IPG) with 4 leads (Fig. 4) supporting for up to 8 channels of current-controlled nerve stimulation (up to ±5mA at 1.6kΩ) and up to 8 channels of biosensing, as described above. The device uses Bluetooth Low Energy for communication with a mobile phone or another external controller (Fig. 4), two rechargeable 200mAh batteries that receive power from a body-worn charger, and I2C connectivity for interfacing with implantable digital sensors, such as an accelerometer. COSMIIC’s system consists of multiple interconnected modules, including those for power, 2-channel EMG sensing, 8-channel kHz stimulation, 64-channel stimulation and sensing, sensing from inertial measurement unit and thermal probe, and a wireless link (last 3 modules are in development) (Makowski 2021; Bender 2026).

### Translational outcomes

The SPARC translational initiative explored new indications for existing devices and funded clinical studies to gain greater mechanistic understanding of targeted neuromodulation therapeutics to serve as a bridge between foundational scientific discoveries and their clinical application.

#### Proof-of-concept: pre-clinical and clinical studies

During Phase 1, the translational initiative supported pre-clinical studies of the safety and efficacy of neuromodulation devices in animal models and humans. In addition to generating clinical data to provide proof of concept, the human studies also involved collection of physiological data beyond the endpoints supporting short-term regulatory milestones to inform functional mapping.

The translational initiative led to many notable developments in Phase 1. At Indiana University, researchers used a canine model of atrial fibrillation, a common and potentially dangerous form of arrhythmia, to compare the effectiveness of vagus nerve stimulation (VNS) and subcutaneous sympathetic nerve stimulation in ameliorating ventricular tachycardia and atrial fibrillation. They found that high-intensity subcutaneous nerve stimulation (ScNS) was more beneficial than VNS in treating atrial fibrillation (Kusayama 2021; Jiang 2018). Low intensity ScNS interestingly showed a proarrhythmic effect that required further investigation (Kusayama 2021). The robust effects observed using high-intensity ScNS (3.5 mA), coupled to their simple “blind” electrode implant procedure that does not require direct contact with a nerve, make this an attractive target for translation to human testing.

A team at the University of Michigan investigated treatments for overactive bladder using a feline model. They compared traditional open-loop sacral nerve stimulation (SNS) with closed-loop SNS, in which bladder sensory signals were recorded from the dorsal root ganglia and used to modulate stimulation in real time. The results showed that closed-loop SNS outperformed open-loop approaches, producing a boost in bladder capacity and a 50+% reduction in the stimulation time needed compared to open-loop stimulation (Ouyang 2022). These results support the development of more efficient and personalized bioelectronics for overactive bladder.

At Johns Hopkins University, a team applied spinal cord stimulation at the thoracic level in a canine model of gastroparesis and observed normalization of gastric motility along with suppression of sympathetic overactivity (Zhang et al. 2019). These findings point to new autonomic communication pathways that might help to further explain the pathophysiology of gastroparesis, while providing new neural targets to explore for bioelectronic regulation of gastric motility disorders. The importance of these findings is underscored by the rarity of treatment-refractory gastroparesis and the lack of any effective long-term pharmaceutical or device-based treatment options. The one device-based treatment option available, the Enterra Gastric Electrical Stimulator, was part of another SPARC-funded project led by Purdue University. The Purdue team and Indiana University School of Medicine, collaborating with BioCircuit Technologies, Inc., validated a new method of measuring vagal compound action potentials through the skin surface of patients implanted with a gastric electrical stimulator for gastroparesis. They found that specific types of response signatures predict improvements in specific types of nausea, vomiting, or other gastroparesis-related symptoms, leading to a patented new method of programming gastric electrical stimulators using vagal nerve response feedback (Ward 2020, 2022; Ward et al. 2016).

The SPARC DRC supported Galvani Bioelectronics and other electroceutical manufacturers in using the o2S2PARC/Sim4Life pipeline (see Technological Innovationssection). It was used to simultaneously optimize neural interface safety and effectiveness (using o2S2PARC’s multi-objective optimization module), select a design for animal testing and model validation, successfully predict differences when translating to human studies, and adapt the interface and stimulation parameters accordingly (Gupta 2020), and produce regulatory evidence. Image processing, AI, (bio)physical and (electro)physiological simulation, and other modules on o^2^S^2^PARC have also been integrated to produce treatment planning tools suitable for clinicians with limited modeling expertise (leveraging o^2^S^2^PARC’s Guided Application Mode). Such tools are successfully deployed and used beyond SPARC’s focus on PNS, e.g., for the personalized planning of brain stimulation (Karimi 2025) and spinal cord stimulation (e.g., for the restoration of locomotion to paraplegics [Rowald 2022]), but also outside the field of neuromodulation (e.g., cancer therapy, MRI implant safety).

Limitations and barriers to translation of these exciting technologies include the need for accurate reliable sensors, long term stability of implanted devices, and the need for robust algorithms to trigger stimulation that can accommodate changing neural outputs in response to varying activities. Preclinical studies occur in highly controlled conditions. Therefore, even after safety is established, translation to investigation in human subjects involves devices ‘in the wild’ in which conditions are far less controlled. Even after human subjects studies demonstrate safety and efficacy, commercialization and general clinical use involve far greater variability in conditions of use and application settings. Nonetheless, these hurdles can be overcome with time, funding, and skilled personnel devoted to the effort. The pursuit of improved technology is well worth investing in despite barriers to translation.

#### A prize-driven push for multi-function selective neuromodulation

In Phase 2, SPARC launched a Neuromodulation Prize Competition with goals of bridging the gap between early-stage research and clinical application of solutions capable of targeting multiple autonomic functions. The competition has rewarded and spurred the development of selective neuromodulation approaches that have the capacity to independently regulate two or more desired autonomic functions without unintended effects. The winning solutions address pressing therapeutic needs, are suitable for or backed by human testing data, enhance or replace existing treatments, and benefit patients and/or practicing clinicians. First-place winner University of Pittsburgh Department of Urology created a multichannel implantable device for sacral-pudendal neuromodulation that addresses bladder, bowel, and sexual disorders. Second-place winner, Juniper Biomedical (formerly RBI Medical), developed a highly precise, micro-implantable neuromodulation system to treat stress urinary incontinence, overactive bladder, and fecal incontinence. Since the goal of translation to clinical use was paramount in awarding the prize money, both winning teams’ devices can be adjusted for individualized treatment, have limited or no side effects, and are now ready to be considered for approval in clinical use.

### Data and modeling infrastructure

**Fig. 5:**
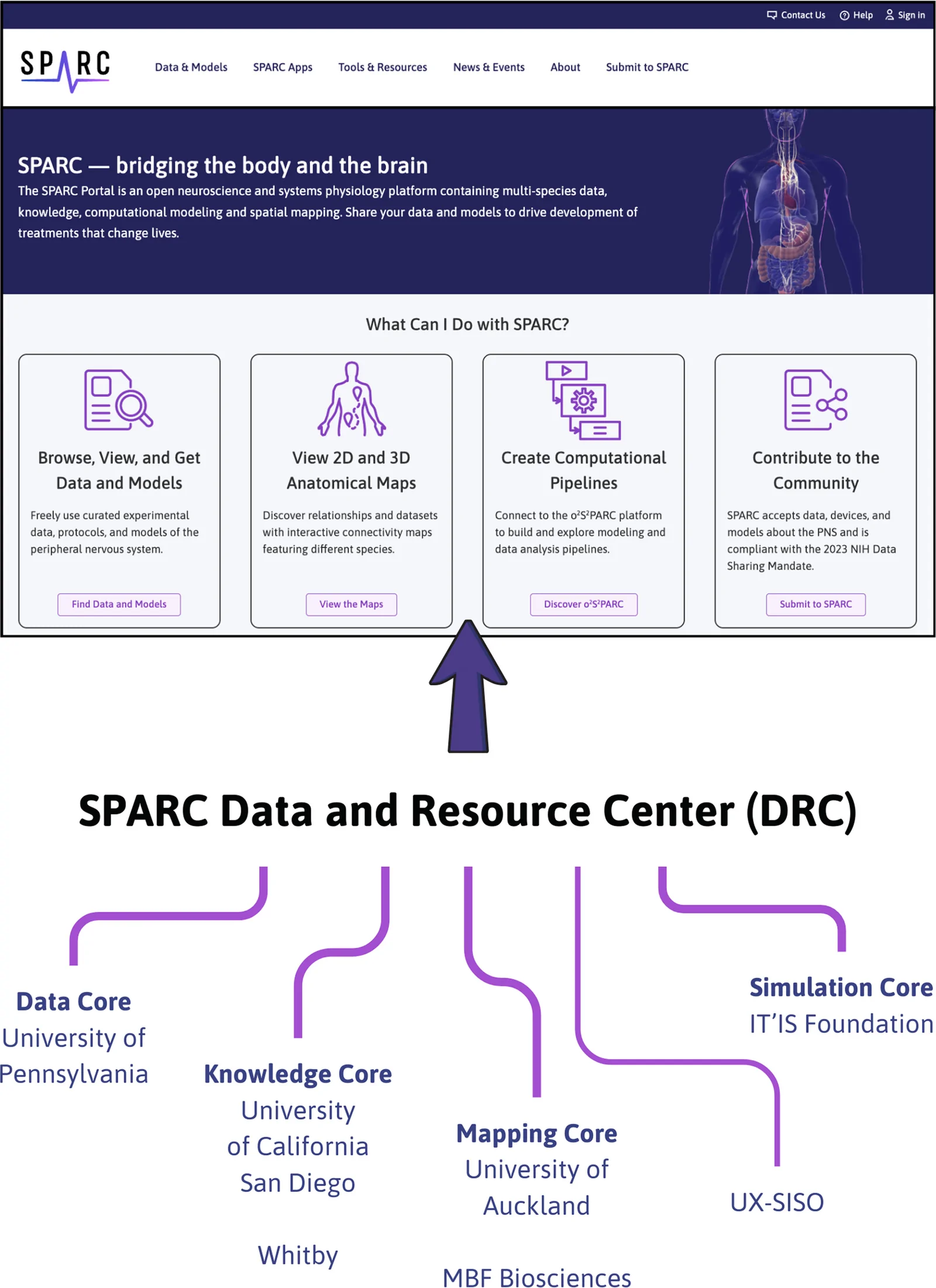
Outcomes from the SPARC Project are freely available on the SPARC Portal. This was developed by the four Cores of the Data and Resource Center (DRC) and shaped with continuous feedback from users

In Phase 1, the SPARC data initiative brought together multiple groups from around the world to form and operationalize the SPARC Data and Resource Center (DRC) (Osanlouy 2021). The DRC is comprised of: 1) The Data Core at The University of Pennsylvania, 2) The Knowledge Core at the University of California San Diego, 3) The Simulation Core at the IT’IS Foundation in Switzerland, and 4) The Mapping Core at The University of Auckland, New Zealand. Together, the DRC established standards and tools for sharing SPARC-funded data, maps, and computational modeling and launched the SPARC Portal (https://sparc.science; Figure 5), where SPARC outcomes are publicly available under permissive reuse licenses. The SPARC Portal is much more than a data repository; it encapsulates a data management platform (Pennsieve), multiple databases and knowledge bases, maps, spatial mapping tools, organ scaffolds, and a computational modeling and analysis platform (o^2^S^2^PARC), along with a host of other viewers and supporting tools oSPARC n.d. ; oSPARC Documentation n.d. ; Osparc-Simcore: Osparc-Simcore Simulation Framework n.d.; Osanlouy 2021; Goldblum 2025). The DRC continues to support the SPARC Portal for Phase 2 projects as these resources make their way onto the Portal. Moreover, the Portal is now an open repository accepting relevant data and models from other funded projects. Investigators looking to satisfy data management and sharing requirements can submit a request to share their research outcomes on the SPARC Portal.

The SPARC Portal differs from many other NIH domain-specific repositories in that it hosts a wide range of data types and models from diverse species and experimental techniques. Several key innovations were made to disseminate these diverse research outcomes in a manner that promotes FAIR meaningful sharing, exploration, understanding, reuse, and collaboration. This includes the development of a specific standard for structuring data and metadata regardless of experimental modality and data type, called the SPARC Data Structure (SDS, current V3) (Bandrowski 2021). Required metadata are outlined in the SPARC Minimal Information Standard (MIS). The SDS (V2) is endorsed by the International Neuroinformatics Coordinating Facility (INCF), making it a trusted community standard for use beyond SPARC. The SDS continues to evolve to support reusability and interoperability.

Meeting the requirements of the SDS entails a high level of compliance by investigators. To help researchers through this process, the DRC embraces a customer-focused service that combines data sharing tools such as the Pennsieve platform, the user-guiding tool SODA (Software to Organize Data Automatically), and human curators (Bandrowski 2021; Marroquin 2023; Patel et al. 2020; Martone 2023). This approach is key to publishing very high-quality data while reducing investigator frustration and errors; moreover, it is also crucial for scaling up and reducing the amount of human curation intervention. SODA has been adapted for use in other projects such as DANDI (DANDI Home n.d.) and Bridge2AI (Nwb-Guide n.d.; BRIDGE2AI n.d.; AI-READI Consortium 2024), and metrics collected over the life of the SPARC project illustrate the value of human curation.

Further exemplifying the value of enforcing the use of the SDS for all data published on the SPARC Portal, is that it supplies the SPARC Knowledge Graph, which allows extensive cross-referencing and interlinking. The obvious links are between datasets and the creators, funding, and source projects. In a similar manner, data mapped to anatomical scaffolds is unambiguously linked back to the source data from which the scaffold map is derived. The Phase 2 REVA data provides a significant test for this capability, with the vagus scaffold maps being derived from many data sources, often with the data sources themselves being compositions of the primary data collected.

In addition to publishing computational models on the SPARC Portal, SPARC offers a dynamic modeling environment hosted through o2S2PARC, an open, online-accessible, cloud-based platform that supports collaborative and FAIR elaboration, sharing, publication, execution, and deployment of simulations, data analysis, expert workflows, and applications (Guidon 2025). It ensures long-term reproducibility and sustainable preservation of computational studies, e.g., by encapsulating computational models and analyses as versioned building-blocks (‘services’) for reusable in larger pipelines. Quality assurance is supported by a testing framework, a computational minimal information standard (cMIS, incorporated into SDS V2), and support for transparency, and adherence to the Ten Simple Rules for Evaluating Model Credibility (10 Simple Rules with Conformance Rubric n.d.; Erdemir 2020).

Expert workflows can be packaged as simple-to-use applications with the o2S2PARC ‘Guided Mode’, for example, to provide treatment planning tools suitable for use by non-expert-modelers. Researchers can easily transform their o2S2PARC models into publishable studies and applications, benefiting from a large number of available modules and from a graphical user interface (GUI). Over 100 services facilitate tasks such as image processing and image-based modeling, mechanistic and data-driven biophysical simulation (e.g., EM, electrophysiology, organ physiology), training and application of deep neural networks, parametrized modeling, control, and interactive data analysis and visualization. Developers can access its powerful API (see https://itisfoundation.github.io/osparc-simcore-clients/#/ and https://api.osparc.io/dev/doc) to perform demanding simulations using the SPARC computational resources. Published modeling application examples include vagus nerve stimulation, organ physiology regulation (e.g., bladder control), compound action potential prediction, multi-scale heart electrophysiology modeling, spike sorting, anatomical scaffolds, and more. o2S2PARC has been applied for therapy personalization (see Translational Outcomes) and is rapidly evolving into a valuable resource for the wider computational life sciences field, with over 2700 registered users (as of February 2026).

Key components of the SPARC infrastructure are open-source projects, and the DRC provides access, documentation, and tutorials for these projects (SPARC sustainability statement n.d.). This framework provides modular, flexible infrastructure and technologies, and can be adapted for other purposes. The DRC is building on this internally by expanding its scope from the ANS to “bridging the body and brain” to support datasets and models involving the broader peripheral nervous system and interactions between the CNS and internal organs.

The decision of the DRC to develop generalized infrastructure that supports multi-modal data and the standardized processes for data curation and publication have made the SPARC data ecosystem deployable for multiple other projects, including the NIH HEAL RE-JOIN, and PRECISION projects (both the SPARC Portal and its underlying data management platform, Pennsieve, are HEAL-compliant repositories), as well as Epilepsy.Science, and The European Vital project. This strategy significantly enhances the sustainability of the resource and provides a pathway towards better integration of the landscape of repositories and knowledgebases in the sciences.

**Fig. 6:**
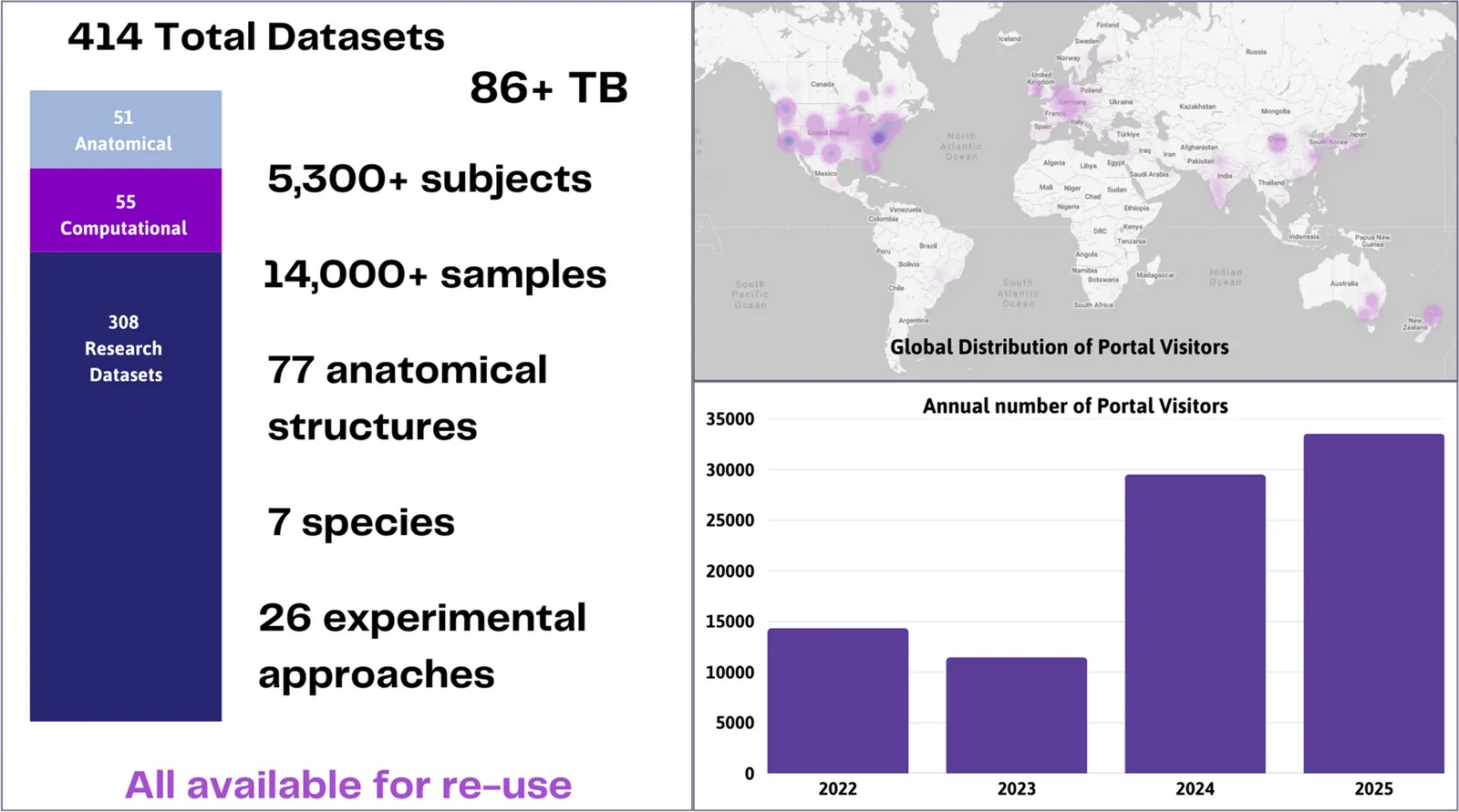
Overview of SPARC Datasets with 414 datasets and over 86 TB published on the SPARC Portal (308 research datasets, 55 computational models, 51 anatomical models). The statistics also show the diverse nature of the data with 77 anatomical structures, 7 species, and 26 experimental approaches represented. Statistics on the left panel are through February 2026, and from December 2025 on the right panel

The rich outcomes offered by the SPARC Program and the impact of the SPARC Portal continue to grow. As of February 2026, a total of 414 datasets and over 86 TB have been published on the SPARC Portal (308 research datasets, 55 computational models, 51 anatomical models). Between February 2023 and December 2025, SPARC datasets have been viewed more than 115,000 times and downloaded more than 15,000 times. During the same period, SPARC models were accessed on o^2^S^2^PARC between 9 to 16 times per day. Over 30 publications have cited SPARC datasets. In addition, 267 protocols linked to these datasets have been shared by SPARC investigators on protocols.io (https://www.protocols.io/workspaces/sparc), which have been viewed more than 50,000 times. Key metrics are summarized in Fig. 6.

## Conclusions

Through strategic investment, targeted initiatives, and cross-disciplinary collaborations, the SPARC program has enhanced the pace of discoveries and innovations in the bioelectronic medicine field since its inception in 2015. The central contribution of the SPARC program is a coordinated pipeline for advancing bioelectronic medicine. The outcomes from the mapping and technology initiatives have supported projects from the translational initiatives, where teams have worked towards clinical validation of existing and novel bioelectronic medicine strategies. These efforts continued in Phase 2 of the SPARC program with an emphasis on the human vagus nerve. Projects supported under the Phase 2 mapping initiative, which are currently wrapping up, are expected to provide a greater understanding of the anatomical organization of the vagus nerve, as well as their functionality and surgical accessibility, to develop new VNS therapies. In parallel, projects supported under the Phase 2 technology and translational initiatives are expected to establish neuromodulation technologies that can target multiple autonomic functions through the vagus nerve. All the outcomes of SPARC-supported projects are shared broadly and openly through the SPARC Portal, which can continue to spur discoveries through secondary use of SPARC data, models, maps, and other outcomes. As a result, the SPARC Portal will continue to contribute towards SPARC’s goal of “catalyzing development of new or more efficacious neuromodulation therapies” in the long term, even after activities related to the SPARC program end.

The impact of the SPARC program goes beyond individual contributions made through these initiatives. SPARC also brought researchers from different disciplines together to advance bioelectronic medicine, a key element in the success of the program. These collaborations are set to continue, as occurred after the end of Phase 1, and will be essential for continuous innovation in the field. SPARC has also set the foundation for a novel data sharing practice in the field through the SPARC Portal, which has expanded its emphasis from the ANS to the PNS and its CNS/end organ interactions. In this way, the SPARC platform provides a necessary complement to the CNS-focused repositories established by the US BRAIN Initiative. The SPARC Portal is now an open community repository serving PNS researchers desiring to share their data and, when applicable, satisfy Data Management and Sharing plans in NIH grants. o^2^S^2^PARC has found adoption beyond the initial autonomic neurosciences field, e.g., in brain stimulation, exposure safety assessment, personalized therapy planning, and medical imaging.

Through novel discoveries, open-source technologies, and open data sharing, SPARC has set the stage for more discoveries and innovations in the field in the years to come. The aim is that bioelectronic medicine will become a widely available, safe, effective, and reliable solution to address chronic diseases and beyond.

## Data Availability

No datasets were generated or analysed during the current study.

## Abbreviations

ANS: Autonomic Nervous System
ASCENT: Automated Simulations to Characterize Electrical Nerve Thresholds
CARSS: Center for Autonomic Recording and Stimulation Systems
CNS: Central Nervous System
ColoMOCA: Colonic Monitor of Conscious Activity
COSMIIC: Cleveland Open Source Modular Implant Innovators Community
DRC: Data and Resource Center
FAIR: Findable, Accessible, Interoperable, Reusable
FDA: Food and Drug Administration
HEAL: Helping to End Addiction Long-term
HORNET: Human Open Research Neural Engineering Technologies
ICNS: Intrinsic Cardiac Nervous System
IEG: Immediate-Early Gene
IPG: Implantable Pulse Generator
MIS: Minimal Information Standard
NIH: National Institutes of Health
PNS: Peripheral Nervous System
REVA: Reconstructing Vagal Anatomy
ScNS: Subcutaneous Nerve Stimulation
SCKAN: SPARC Connectivity Knowledge Base
SDS: SPARC Data Structure
SNS: Sacral Nerve Stimulation
SPARC: Stimulating Peripheral Activity to Relieve Conditions
UroMOCA: Urological Monitor of Conscious Activity
USA: United States of America
VESPA: VNS Endpoints from Standardized Parameters
VNS: Vagus Nerve Stimulation
